# Epidemiology and Economic Burden of Continuing Challenge of Infectious Diseases in India: Analysis of Socio-Demographic Differentials

**DOI:** 10.3389/fpubh.2022.901276

**Published:** 2022-06-30

**Authors:** Bhed Ram, Ramna Thakur

**Affiliations:** School of Humanities and Social Sciences (SHSS), Indian Institute of Technology Mandi, Kamand, India

**Keywords:** infectious diseases, out-of-pocket expenditure, source of finance, coping, low- and middle-income countries

## Abstract

Unlike other low- and middle-income countries, infectious diseases are still predominant, and non-communicable diseases (NCDs) are emerging without replacing the burden of infectious diseases in India, where it is imposing a double burden of diseases on households in the country. This study aimed to analyse the socio-economic and demographic differentials in the magnitude of economic burden and coping strategies associated with health expenditure on infectious diseases in India. National Sample Survey Organization (NSSO) data on “Key Indicators of Social Consumption in India: Health, (2017–18)” have been employed in this study. The findings of the study revealed that more than 33% of the individuals are still suffering from infectious diseases out of the total ailing population in India. Based on the various socio-economic and demographic covariates, infectious diseases are highly prevalent among individuals with marginalized characteristics, such as individuals residing in rural areas, females, 0–14 age groups, Muslims, illiterates, scheduled tribes (STs), and scheduled castes (SCs), large family households, and economically poor people in the country. The per capita out-of-pocket (OOP) expenditure on infectious diseases is INR 7.28 and INR 29.38 in inpatient and outpatient care, respectively. Whereas, monthly per patient OOP expenditure on infectious diseases by infection-affected populations is INR 881.56 and INR 1,156.34 in inpatient and outpatient care in India. The study found that people residing in rural areas, SCs followed by other backward classes (OBCs), illiterates, poor, and very poor are more dependent on borrowings, sale of assets, and other distressed sources of financing. However, under National Health Policy 2017, many initiatives, such as “Ayushman Bharat,” PM-JAY, and National Digital Health Mission (NDHM) in 2021, have been launched by the government of India in the recent years. These initiatives are holistically launched for ensuring better health facilities, but it is early to make any prediction regarding its outcomes; hopefully, the time will define it over the passing of a few more years. Finally, the study proposed the need for proper implementations of policy initiatives, awareness against unhygienic conditions and contamination of illnesses, immunisations/vaccination campaigns, subsidized medical facilities, and the country's expansion of quality primary health-care facilities.

## Introduction

The narratives of public health are facing a significant challenge in demographic and epidemiological transitions, particularly in low- and middle-income countries (LMICs) (1–3). This transition has changed the pattern and distribution of morbidity and mortality among inhabitants and exaggerated the burden on these countries' pre-existing inadequate public health systems (1). Although several life-threatening diseases have been cured through various preventive, curative, and policy measures, infectious diseases are still one of the leading causes of death in LMICs (4, 5). These are the diseases caused by pathogenic microorganisms, such as viruses, bacteria, protozoa, parasites, and fungi. It spreads through direct or indirect interaction with unhygienic conditions (6, 7). Adverse living conditions are expected in LMICs, further augmenting the number and severity of infectious diseases in these countries (7–11).

World Health Organization (WHO) has ranked infections, such as lower respiratory infections, diarrheal diseases, and tuberculosis, in the top 10 causes of mortality worldwide in 2016. Most of the burden of these diseases has been observed in LMICs (12, 13). HIV/AIDS is still a significant cause of death in LMICs but left out from the list of top ten causes of death from 2000 to 2016 worldwide (13). Similarly, malaria prevalence has been reduced due to massive investment and policy initiatives in the past years. However, eliminating malaria is still a big challenge in most LMICs (14). New infectious diseases, such as avian flu, swine flu, and coronavirus, are also emerging at a higher rate and spreading more rapidly than ever in the communities (15, 16).

India is not an exception in such a transition. However, unlike other LMICs, infectious diseases are still predominant, and non-communicable diseases (NCDs) are emerging without replacing the country's burden of contagious diseases (17, 18). This double burden of diseases is a serious public health concern where NCDs are continuously increasing with prevalent contagious diseases, a significant cause of premature mortality among people in the country (17–21). Various long-standing infectious diseases, such as smallpox, guinea worm, polio, leprosy, cholera, and plague, have been controlled and eradicated from the community. However, diseases, such as dengue, malaria, typhoid, and tuberculosis, are still the common causes of febrile illness among people in India (17, 22, 23). In tuberculosis, India is the top country globally, with a 26% share of this disease in the global burden of diseases (13). After South Africa, India is the leading country suffering from HIV/AIDS worldwide, where 4.6 million people are infected with this life-threatening disease (13, 24, 25). Even among neonatal, the frequency of infectious diseases, such as typhoid, diarrhea, measles, tuberculosis, and jaundice, remains the primary cause of infant morbidity and mortality in India. The findings from various studies show that the burden of NCDs has increased from 37.9 to 61.8%, whereas the burden of communicable and infectious diseases is still at 27.5% for three decades in the country (21, 26).

Similarly, in a study, Banerjee and Dwivedi (27) observed that the prevalence of infectious diseases had slightly reduced between 2004 and 2014. But it is still stagnant and has become a significant challenge to public health. However, Paul and Singh (28) observed that the prevalence of infectious diseases in outpatient care increased nearly three times, from 8 to 26 per 1,000 in two decades (1995 to 2014). Also, typhoid fever incidence has been reported at 27.3 at the age under 5 years, 11.7 at 5 to 19 years, and 1.1 between 19 and 40 years per 1,000 person among residents of a low-income urban area of Delhi, India (29).

It is clear from the above discussion that infectious diseases are still contributing to both the physical and the economic burden of diseases in India (30). However, the Indian economy is one of the fastest-growing economies globally with a 6–7% annual average gross domestic product (GDP) growth rate but spends only 1%of its GDP on health (31, 32). Insufficient health insurance coverage also plays a crucial role in contributing to the country's increasing economic burden of diseases (33). This meager amount of public health spending and under-coverage of health insurance increases the household's dependence on out-of-pocket expenditure. The households' inability to cope with the economic burden of diseases pushes them into poverty (34–36).

Differences and vulnerabilities based on the socio-economic characteristics are common phenomena among the Indian population. Due to these vulnerabilities, the emergence and re-emergence of infectious diseases have been consistent among inhabitants for centuries. However, many contagious diseases have not even been controlled but also have been eliminated from society. Despite it, many of them are still more susceptible to the population in the country. Also, these diseases do not harm only individuals' health status but also impose an economic burden. A few studies, such as Visaria (21), Banerjee and Dwivedi (27), and Paul and Singh (28), have conducted an analysis of infectious diseases using nationally representative sample survey data but explored only the prevalence and its association with background characteristics. These studies lack to analyse the economic burden, especially out-of-pocket expenditure and finance's primary source, to cope with spending on infectious diseases. Considering the importance of the associated socio-economic covariates and economic dimensions compels us to analyse the problem accordingly. Hence, the study aims to analyse the prevalence and financial burden of out-of-pocket expenditure and source of finance on infectious diseases among various socio-economic and demographic covariates in India. Also, it is expected that the results of this study would provide a deep insight into the problem, which will work as a roadmap for the policymakers to control the same through appropriate policy initiatives.

## Methodology

### Data

The analysis is based on cross-sectional data from the National Sample Survey Organization (NSSO), 75th Round (2017–2018) on Key Indicators of Social Consumption in India: Health (37). The survey consists of a sample of 113,823 households comprising 555,115 individuals. In inpatient care, 19,443 individuals, out of 58,214 ailments affected sample persons, reported suffering from at least any infectious diseases during the recall period of 365 days. While on outpatient care, 10,960 individuals, out of 39,778 ailments affected sample persons, reported suffering from at least any infectious conditions during 15-day recall period. Furthermore, all contagious diseases have been analyzed collectively as infectious diseases both in inpatient and outpatient care in India.

### Methods

In this study, the prevalence (P_i_) of infectious diseases has been calculated as follows: $Pi=\;\frac{1}{N}\overset{n}{\underset{i=1}{\sum }}I$, where “*N*” is the population size and “*I*” is the number of individuals affected by infectious diseases.

The economic burden of infectious diseases among various socio-economic covariates has been measured in terms of out-of-pocket expenditure on infectious diseases as a percentage share of total consumption expenditure (TCE) is given by: $OOP_{TCE}=\sum_{i=1}^{n}OOP_{i}/\sum_{i=1}^{n}MPCE_{i}\times 100$, where “*OOP*_(*TCE*)_” stands for out-of-pocket expenditure as a percentage share of total consumption expenditure, “*OOP*_*i*_” is the out-of-pocket expenditure on infectious diseases, and “*MPCE*_*i*_” is the monthly per capita consumption expenditure of the ith individual.

While out-of-pocket expenditure on infectious diseases as a percentage share of total consumption expenditure (TCE) of a country's infection affected population at different threshold levels (reporting level, 5, 10, and 15%) is given by $OOP_{TCE_{iap}}=\sum_{i=1}^{n}OOP_{i}/\sum_{i=1}^{n}MPCE_{iap}\times 100$, where “*OOP*_*TC*_*E*__*iap*__” is the out-of-pocket expenditure on infectious diseases and “*MPCE*_*iap*_” is the monthly per capita consumption expenditure of the country's infection-affected population.

Also, the average per capita OOP health expenditure on the infection has been measured as; *Per Capita OOP* $=\sum_{i=1}^{n}OOP_{i}/N$, where “*OOP*_*i*_” is the out-of-pocket expenditure on infectious diseases and “*N*” denotes the total population. Furthermore, average per capita OOP expenditure on infection by the individuals particularly suffering from infectious diseases has been measured as; *Per Patient* $OOP=\sum_{i=1}^{n}OOP_{i}/\sum_{i=1}^{n}$*Q*, where “*OOP*_*i*_” is the out-of-pocket expenditure on infectious diseases and ‘*Q'* is the number of people affected with infectious diseases.

Finally, the source of finance to cope with OOP expenditure on infectious diseases has also been measured. Savings/income, borrowings, contributions from friends and relatives, sale of physical assets, and other resources have been taken as the individuals' key strategies or sources of finance. In the case of inpatient care, information on coping strategies has been given as the first and second primary sources of finance. While on outpatient care, the only first significant source of finance has been shown in the data source. Therefore, to ensure the similarities in inpatient and outpatient care, only the first important source of finance has been taken in the analysis. Besides the critical coping strategies, such as savings/income and borrowings, the remaining basis of finance has been taken collectively due to their less significant share and simplification of the analysis. The percentage of different sources of finance used to cope with the OOP health expenditure on the infection has been calculated as $Y=\sum_{i=1}^{n}U/V*100$.

where “Y” is the percentage share of a source of finance, “U” is the source of finance, and “V” is the sum of all sources of finance.

### Variables of the Study

#### Dependent Variable

The study's dependent variable is morbidity due to infectious diseases among inpatient and outpatient care individuals.

#### Independent Variable

In this analysis, the independent variables are the place of residence (rural/urban), sex (male, female, and transgender), age (0–14, 15–29, 30–59, and >60), education (illiterate, up to primary, secondary and graduation, and above), religious groups (Hindu, Muslims, and others), social groups (scheduled tribes, scheduled castes, other backward classes, and others), economic status (wealth quintiles, such as very poor, poor, average, rich, and very rich), households size (less than average and more than average), and region (north, north-east, east, central, west, and south). In Indian society, religion is one of the critical variables broadly divided into Hindus, Muslims, Sikhs, Buddhism, Christianity, Jainism, Zoroastrianism, and many smaller religious groups. But in this analysis, we have classified religion into three major categories, *viz*. Hindus, Muslims, and the remaining have been included in other religious groups because of their small size in the total population. The monthly per capita consumption expenditure (MPCE) has been taken as a proxy for income to measure the economic status of the individuals. It has been ranked from very poor to a very rich one. The analysis categorizes households' sizes into less than average and more than average. Furthermore, all states and union territories have been characterized into six regions based on their geographical locations: north, north-east, east, central, west, and south.

## Results of the Study

### Summary Statistics

The details of sample persons and the respective estimated population in different demographic and socio-economic categories, i.e., place of residence, sex, age, religion, social classification, economic status, etc., have been given in Table 1. A total of 555,115 sample persons have been surveyed, representing the 1,140,187,554 total population of the country. Individuals suffering from various ailments have been shown in two categories, i.e., inpatient and outpatient. Where 58,214 individuals reported suffering from multiple diseases and were hospitalized during the last 365 days before the day of the survey, 19,443 individuals stated that they were affected by infectious ailments during this time. While on 15-day recall period, a sample of 39,778 persons reported suffering from various diseases, out of which only 10,960 were affected with infectious diseases during this period.

**Table 1:** Summary statistics of the sample population.

**Variables**	**Estimated and sample population**	**Inpatient**		**Outpatient**	
		**Total ailing population**	**Population ailing from infectious diseases**	**Total ailing population**	**Population ailing from infectious diseases**
All India	1,140,187,554	27,783,232	9,419,082	85,269,522	28,969,799
	(555,115)	(58,214)	(19,443)	(39,778)	(10,960)
Place of residence					
Rural	804,273,325	17,989,881	6,041,486	54,781,294	20,571,709
	(325,883)	(32,441)	(11,048)	(20,802)	(6,506)
Urban	335,914,229	9,793,351	3,377,596	30,488,228	8,398,090
	(229,232)	(25,773)	(8,395)	(18,976)	(4,454)
Sex					
Male	589,257,319	14,075,161	4,833,223	39,474,970	14,352,788
	(283,200)	(30,033)	(10,076)	(18,948)	(5,372)
Female	550,864,001	13,704,591	4,584,209	45,793,674	14,617,011
	(271,878)	(28,177)	(9,365)	(20,829)	(5,588)
Transgender	66,234	3,480	1,650	878	0
	(37)	(4)	(2)	(1)	0
Age					
0–14	301,230,284	4,676,254	2,827,072	17,561,555	12,063,353
	(155,647)	(10,208)	(6,322)	(7,946)	(5,162)
15–29	318,486,584	5,198,084	1,996,661	10,555,762	5,921,053
	(152,909)	(10,750)	(4,215)	(4,316)	(2,044)
30–59	441,153,086	12,628,168	3,626,929	35,162,767	8,485,415
	(203,797)	(26,185)	(7,032)	(15,746)	(2,792)
60+	79,317,600	5,280,726	968,420	21,989,438	2,499,978
	(42,762)	(11,071)	(1,874)	(11,770)	(962)
Religion					
Hindu	925,019,675	22,107,923	7,399,669	66,622,950	22,844,158
	(412,512)	(44,026)	(14,485)	(29,273)	(8,089)
Muslim	161,096,251	3,939,529	1,436,344	13,039,977	4,512,422
	(83,001)	(7,805)	(2,534)	(6,750)	(1,901)
Others*	54,071,628	1,735,780	583,069	5,606,595	1,613,219
	(59,602)	(6,383)	(2,424)	(3,755)	(970)
Social groups					
STs	103,490,979	1,710,964	658,676	5,221,281	2,421,788
	(75,256)	(6,885)	(2,998)	(2,783)	(1,079)
SCs	223,840,510	5,296,687	1,824,955	15,555,824	5,955,188
	(94,062)	(9,621)	(3,228)	(6,502)	(bib2,046)
OBCs	512,112,220	12,169,154	4,191,417	36,144,771	13,170,645
	(222,766)	(23,369)	(7,713)	(15,943)	(4,648)
Others	300,743,845	8,606,427	2,744,034	28,347,646	7,422,178
	(163,031)	(18,339)	(5,504)	(14,550)	(3,187)
General education					
Illiterate	297,266,691	8,996,019	3,028,885	30,587,274	10,580,025
	(147,275)	(17,117)	(5,694)	(14,156)	(4,503)
Up to primary	498,126,420	11,108,187	3,989,067	34,039,172	12,678,283
	(226,320)	(23,076)	(8,429)	(15,038)	(4,246)
Secondary	250,785,824	5,703,816	1,819,081	14,596,197	4,314,580
	(126,888)	(12,880)	(3,946)	(7,260)	(1,646)
Graduation and above	94,008,619	1,975,210	582,049	6,046,879	1,396,911
	(54,632)	(5,141)	(1,374)	(3,324)	(565)
Wealth quintile					
Very poor	534,658,598	9,524,508	3,358,934	30,175,229	14,049,087
	(218,202)	(18,152)	(6,414)	(10,970)	(4,309)
Poor	268,007,766	6,975,140	2,318,274	19,937,428	6,601,036
	(134,525)	(13,823)	(4,616)	(9,342)	(2,709)
Average	164,381,344	5,179,085	1,764,899	15,379,224	4,368,359
	(97,343)	(11,484)	(3,869)	(8,123)	(1,966)
Rich	107,479,414	3,857,699	1,301,897	12,010,674	2,613,975
	(67,558)	(8,777)	(2,797)	(6,785)	(1,312)
Very rich	65,660,432	2,246,800	675,078	7,766,967	1,337,342
	(37,487)	(5,978)	(1,747)	(4,558)	(664)
Households size**					
Less than average	687,672,512	18,922,107	6,515,377	57,874,634	18,547,100
	(289,829)	(39,646)	(13,530)	(23,870)	(6,173)
More than average	452,515,042	8,861,125	2,903,705	27,394,888	10,422,699
	(265,286)	(18,568)	(5,913)	(15,908)	(4,787)
Regions***					
North	160,836,817	3,768,445	1,174,847	10,385,958	3,809,508
	(105,232)	(10,349)	(3,030)	(7,187)	(2,009)
Northeast	44,044,744	658,172	259,272	1,025,085	490,600
	(72,324)	(7,129)	(2,926)	(1,436)	(581)
Central	284,261,422	5,362,634	1,923,078	18,086,039	9,075,577
	(106,814)	(9,520)	(3,156)	(6,416)	(2,497)
East	250,025,083	5,526,488	1,645,190	19,914,913	6,697,091
	(94,334)	(9,632)	(2,811)	(7,649)	(2,269)
West	159,468,994	4,116,808	1,471,742	12,813,455	4,201,608
	(68,771)	(7,457)	(2,631)	(5,313)	(1,374)
South	241,550,494	8,350,685	2,944,953	23,044,072	4,695,415
	(107,640)	(14,127)	(4,889)	(11,777)	(2,230)

*Figures are based on the author's calculations from the NSSO 75th rounds*.

*Values in parentheses are sample sizes*.

^*^Include remaining religions such as Christianity, Sikhism, Jainism, Buddhism, Zoroastrianism, and others.

^**^Households size is categorized into two categories viz. less than average and more than average.

^***^North: J&K, Himachal Pradesh, Punjab, Chandigarh, Uttarakhand, Haryana, Delhi, Rajasthan; Northeast: Sikkim, Arunachal Pradesh, Nagaland, Manipur, Mizoram, Tripura, Meghalaya, Assam; East: Bihar, West Bengal, Jharkhand, Odisha; Central: Utter Pradesh, Chhattisgarh, Madhya Pradesh; West: Gujrat, Daman, and Diu, Dadar and Nagar Haveli, Maharashtra, Goa; South: Andhra Pradesh, Karnataka, Lakshadweep, Kerala, Tamil Nadu, Pondicherry, Andaman and Nicobar, Telangana.

### Prevalence Rate and the Proportion of Population Spending on Infectious Diseases

Table 2 shows that out of 1,000 persons, 8.26 persons reported infectious diseases in inpatient care, which is 33.90% of the country's total ailing population. Furthermore, the percentage share of population spending and their income regarding their total consumption expenditure (TCE) on infectious diseases has been measured at different threshold levels. The finding shows that nearly 0.66, 0.54, and 0.45% of the population suffering from infections are spending more than 5, 10, and 15% of their TCE on infectious diseases in India. The analysis shows a significant variation among demographic and socio-economic covariates in the prevalence of infectious diseases. Therefore, the association of several demographic and socio-economic variables with infectious diseases has been analyzed in the study. The prevalence is highest among individuals residing in the urban area (10.05 individuals), among transgender people (24.92 individuals), 60+ age group (12.21 individuals), other religious groups (10.78 individuals), other social groups (9.12 individuals), illiterate people (10.19 individuals), and people coming from the southern part of the country (12.19 individuals) than their respective correspondents in the study.

**Table 2:** Socio-economic and demographic covariates in the prevalence rate (out of 1,000), and the population spending OOP as a percentage of total consumption expenditure (TCE) on infectious diseases at various thresholds levels in India (2017–18).

**Variables**	**Inpatient**					**Outpatient**				
	**Prevalence of infectious diseases affected population (out of 1,000)**	**Percentage of individuals suffering from infectious diseases out of total ailing population**	**Population spending more than 5% of TCE on infectious diseases** ^**#**^	**Population spending more than 10% of TCE on infectious diseases** ^**#**^	**Population spending more than 15% of TCE on infectious diseases** ^**#**^	**Prevalence of infectious diseases affected population (out of 1,000)**	**Percentage of individuals suffering from infectious diseases out of total ailing population**	**Population spending more than 5% of TCE on infectious diseases** ^**#**^	**Population spending more than 10% of TCE on infectious diseases** ^**#**^	**Population spending more than 15% of TCE on infectious diseases** ^**#**^
All India	8.26	33.90	7,566,479	6,206,458	5,118,207	25.41	33.97	24,313,160	21,028,176	18,607,805
			(0.66)	(0.54)	(0.45)			(2.13)	(1.84)	(1.63)
Place of residence										
Rural	7.51	33.58	4,908,874	4,109,357	3,458,224	25.58	37.55	17,686,381	15,389,695	13,795,506
			(0.61)	(0.51)	(0.43)			(2.20)	(1.91)	(1.72)
Urban	10.05	34.49	2,657,605	2,097,101	1,659,982	25.00	27.55	6,626,779	5,638,481	4,812,299
			(0.79)	(0.62)	(0.49)			(1.97)	(1.68)	(1.43)
Sex										
Male	8.20	34.34	3,928,688	3,195,816	2,647,006	24.36	36.36	11,958,945	10,497,358	9,473,398
			(0.67)	(0.54)	(0.45)			(2.03)	(1.78)	(1.61)
Female	8.32	33.45	3,636,635	3,009,487	2,470,045	26.53	31.92	12,354,215	10,530,818	9,134,407
			(0.66)	(0.55)	(0.45)			(2.24)	(1.91)	(1.66)
Transgender	24.92	47.42	1,156	1,156	1,156	0	0	0	0	0
			(1.74)	(1.74)	(1.74)					
Age										
0–14	9.39	60.46	2,355,396	1,935,175	1,577,076	40.05	68.69	10,651,752	9,361,453	8,424,760
			(0.78)	(0.64)	(0.52)			(3.54)	(3.11)	(2.80)
15–29	6.27	38.41	1,593,614	1,297,719	1,097,511	18.59	56.09	4,834,563	4,152,473	3,648,294
			(0.50)	(0.41)	(0.34)			(1.52)	(1.30)	(1.15)
30–59	8.22	28.72	2,848,611	2,320,435	1,910,599	19.23	24.13	6,762,962	5,781,272	4,988,549
			(0.65)	(0.53)	(0.43)			(1.53)	(1.31)	(1.13)
60+	12.21	18.34	768,858	653,128	533,020	31.52	11.37	2,063,884	1,732,978	1,546,202
			(0.97)	(0.82)	(0.67)			(2.60)	(2.18)	(1.95)
Religion										
Hindu	8.00	33.47	5,988,086	4,943,440	4,066,503	24.70	34.29	19,296,804	16,804,927	14,905,312
			(0.65)	(0.53)	(0.44)			(2.09)	(1.82)	(1.61)
Muslim	8.92	36.46	1,116,550	902,428	759,880	28.01	34.60	3,723,305	3,156,296	2,792,440
			(0.69)	(0.56)	(0.47)			(2.31)	(1.96)	(1.73)
Others*	10.78	33.59	461,843	360,589	291,823	29.83	28.77	1,293,052	1,066,953	910,053
			(0.85)	(0.67)	(0.54)			(2.39)	(1.97)	(1.68)
Social groups										
STs	6.36	38.50	498,904	392,379	316,222	23.40	46.38	1,914,754	1,667,578	1,485,687
			(0.48)	(0.38)	(0.31)			(1.85)	(1.61)	(1.44)
SCs	8.15	34.45	1,403,728	1,056,581	892,188	26.60	38.28	4,841,682	4,171,069	3,606,080
			(0.63)	(0.47)	(0.40)			(2.16)	(1.86)	(1.61)
OBCs	8.18	34.44	3,430,279	2,893,105	2,397,502	25.72	36.44	11,261,062	9,731,786	8,657,122
			(0.67)	(0.56)	(0.47)			(2.20)	(1.90)	(1.69)
Others	9.12	31.88	2,233,568	1,864,393	1,512,295	24.68	26.18	6,295,662	5,457,743	4,858,916
			(0.74)	(0.62)	(0.50)			(2.09)	(1.81)	(1.62)
General education										
Illiterate	10.19	33.67	2,449,347	2,038,858	1,699,493	35.59	34.59	9,037,526	7,928,451	6,948,430
			(0.82)	(0.69)	(0.57)			(3.04)	(2.67)	(2.34)
Up to primary	8.01	35.91	3,158,874	2,508,953	2,048,849	25.45	37.25	10,695,820	9,245,178	8,195,519
			(0.63)	(0.50)	(0.41)			(2.15)	(1.86)	(1.65)
Secondary	7.25	31.89	1,482,428	1,260,815	1,031,526	17.20	29.56	3,496,711	2,928,393	2,644,182
			(0.59)	(0.50)	(0.41)			(1.39)	(1.17)	(1.05)
Graduation and above	6.19	29.47	475,829	397,832	338,338	14.86	23.10	1,083,103	926,154	819,673
			(0.51)	(0.42)	(0.36)			(1.15)	(0.99)	(0.87)
Wealth quintile					
Very poor	6.28	35.27	2,868,802	2,404,820	2,074,478	26.28	46.56	12,116,112	10,500,884	9,350,584
			(0.54)	(0.45)	(0.39)			(2.27)	(1.96)	(1.75)
Poor	8.65	33.24	1,784,914	1,488,153	1,268,611	24.63	33.11	5,587,556	5,043,970	4,444,407
			(0.67)	(0.56)	(0.47)			(2.08)	(1.88)	(1.66)
Average	10.74	34.08	1,372,420	1,084,720	864,461	26.57	28.40	3,621,808	3,063,676	2,688,672
			(0.83)	(0.66)	(0.53)			(2.20)	(1.86)	(1.64)
Rich	12.11	33.75	1,044,103	855,777	630,876	24.32	21.76	2,007,087	1,655,098	1,447,385
			(0.97)	(0.80)	(0.59)			(1.87)	(1.54)	(1.35)
Very rich	10.28	30.05	496,240	372,989	279,780	20.37	17.22	980,597	764,548	676,756
			(0.76)	(0.57)	(0.43)			(1.49)	(1.16)	(1.03)
Household size**					
Less than average	9.47	34.43	5,088,301	4,092,923	3,272,155	26.97	32.05	14,638,243	12,229,554	10,479,367
			(0.74)	(0.60)	(0.48)			(2.13)	(1.78)	(1.52)
More than average	6.42	32.77	2,478,178	2,113,536	1,846,052	23.03	38.05	9,674,918	8,798,622	8,128,438
			(0.55)	(0.47)	(0.41)			(2.14)	(1.94)	(1.80)
Regions***					
North	7.30	31.18	946,173	725,453	616,859	23.69	36.68	3,231,769	2,764,717	2,491,067
			(0.59)	(0.45)	(0.38)			(2.01)	(1.720	(1.55)
Northeast	5.89	39.39	228,147	162,731	115,253	11.14	47.86	367,352	334,918	312,119
			(0.52)	(0.37)	(0.26)			(0.83)	(0.76)	(0.71)
East	6.77	35.86	1,650,676	1,447,197	1,242,895	31.93	50.18	8,022,211	6,829,588	6,044,282
			(0.58)	(0.51)	(0.44)			(2.82)	(2.40)	(2.13)
Central	6.58	29.77	1,252,856	982,828	774,223	26.79	33.63	5,645,658	5,088,658	4,658,459
			(0.50)	(0.39)	(0.31)			(2.26)	(2.04)	(1.86)
West	9.23	35.75	1,215,744	1,050,639	891,142	26.35	32.79	3,361,856	2,945,451	2,565,950
			(0.76)	(0.66)	(0.56)			(2.11)	(1.85)	(1.61)
South	12.19	35.27	2,272,883	1,837,610	1,477,834	19.44	20.38	3,684,314	3,064,844	2,535,928
			(0.94)	(0.76)	(0.61)			(1.53)	(1.27)	(1.05)

*Figures are based on the author's calculations from the NSSO 75th rounds*.

*^#^Values in parentheses show the percentage share of the total population*.

*Include remaining religions such as Christianity, Sikhism, Jainism, Buddhism, Zoroastrianism, and others.

**Households size is categorized into two categories viz. less than average and more than average.

***North: JandK, Himachal Pradesh, Punjab, Chandigarh, Uttarakhand, Haryana, Delhi, Rajasthan; Northeast: Sikkim, Arunachal Pradesh, Nagaland, Manipur, Mizoram, Tripura, Meghalaya, Assam; East: Bihar, West Bengal, Jharkhand, Odisha; Central: Utter Pradesh, Chhattisgarh, Madhya Pradesh; West: Gujrat, Daman, and Diu, Dadar and Nagar Haveli, Maharashtra, Goa; South: Andhra Pradesh, Karnataka, Lakshadweep, Kerala, Tamil Nadu, Pondicherry, Andaman and Nicobar, Telangana.

Similarly, in outpatient care, the prevalence has been reported at 25.41 persons out of 1,000 persons in India and is 33.97% of the country's total ailing population. Furthermore, the findings reveal that instead of their absolute numbers, nearly 2.13, 1.84, and 1.63% population suffering from infections are spending more than 5, 10, and 15% of their TCE on infectious diseases in India. Whereas, based on various socio-economic and demographic variables, the prevalence and percentage of people suffering from infectious diseases is highest among people residing in rural areas (25.58 individuals), among females (26.53 individuals), among 0–14 age groups (40.04 individuals), among other religious groups (29.83 individuals), more than average family size (26.97), among SCs (26.60 individuals), among the central region of the country (31.93 individuals) than their corresponding variables in the analysis. Also, the proportion of people suffering from infectious diseases out of the total ailing population is highest among people residing in the rural area, male, 0–14 age group, Muslims, scheduled tribes (STs), education up to secondary level, very poor economic status, large family size, and northern regions of the country. However, a similar trend has been found in the percentage and prevalence of infection-affected populations among various socio-economic and demographic variables. Still, it differs in the case of absolute numbers of people suffering from infections and the population spending OOP expenditure equal to their TCE at various threshold levels in the study. The analysis shows that the dependent age groups, such as 60+ and 0–14, are more suffering from infectious diseases. While education emerges as a critical preventive factor, an increase in the number of years spent in an educational institution positively impacts the dominance of infectious diseases in communities. Further, the prevalence of infectious diseases is growing, with an increase in India's economic level. In comparison to the emergence of the ailments, it is describing the ability to report and access healthcare facilities by the economically sound section of the society in India. Indeed, these figures confirm the notion that people belonging to low-income families may not be able to hospitalize their family members during severe life-threatening ailments due to their insufficient financial resources or low economic status in India.

### Level of Out-of-Pocket Expenditure on Infectious Diseases

Table 3 shows that in the case of inpatient care, the overall average per capita OOP expenditure on infectious diseases is INR 7.28 in the country. Whereas, for various socio-economic and demographic variables, it is highest among urban areas (INR 10.42), among transgender people (INR 8.57), among 60+ age groups (INR 11.64), among other religious groups (INR 9.35), among different social groups (INR 10.34), among educated up to secondary level (INR 8.98), among economically rich people (INR 15.38), among less than average households (INR 7.98), and people populated in the southern region (INR 9.72) of India. Furthermore, we have also calculated the average monthly per capita OOP expenditure of infection-affected population. Results show that, on average, INR 881.56 has been spent per month on inpatient care in India. The distinction between various socio-economic and demographic covariates has been found in the analysis. The average monthly per capita OOP expenditure on infectious diseases is relatively low among people residing in rural areas, transgender people, Muslims, STs, people educated up to primary level and illiterates, economically poor, and people from the north-east region of the country.

**Table 3:** Socio-economic and demographic covariates in the level of out-of-pocket (OOP) expenditure as a share of total consumption expenditure (TCE) on infectious diseases in India (2017–18).

**Variables**	**Inpatient**							**Outpatient**						
	**Average per capita OOP expenditure on infectious diseases (INR)**	**OOP expenditure on infectious diseases as a percentage of TCE (%)**	**Per capita OOP expenditure of the individuals suffering from infectious diseases (INR)**	**OOP expenditure as a percentage of TCE of the individuals suffering from infectious diseases** ***(% at reporting level)***	**OOP health expenditure as a percentage of TCE of the individuals suffering from infectious diseases** ***(5%)***	**OOP health expenditure as a percentage of TCE of the individuals suffering from infectious diseases** ***(10%)***	**OOP health expenditure as a percentage of TCE of the individuals suffering from infectious diseases** ***(15%)***	**Average per capita OOP expenditure on infectious diseases (INR)**	**OOP expenditure on infectious diseases as a percentage of TCE (%)**	**Per capita OOP expenditure of the individuals suffering from infectious diseases (INR)**	**OOP expenditure as a percentage of TCE of the individuals suffering from infectious diseases** ***(% at reporting level)***	**OOP health expenditure as a percentage of TCE of the individuals suffering from infectious diseases** ***(5%)***	**OOP health expenditure as a percentage of TCE of the individuals suffering from infectious diseases** ***(10%)***	**OOP health expenditure as a percentage of TCE of the individuals suffering from infectious diseases** ***(15%)***
All India	7.28	0.34	881.56	36.04	31.59	28.06	25.23	29.38	1.06	1156.34	35.82	31.74	28.70	26.26
Place of residence														
Rural	5.97	0.36	794.98	42.50	37.99	34.39	31.41	28.01	1.29	1095.23	44.16	39.93	36.59	33.83
Urban	10.42	0.31	1036.43	29.83	25.42	21.96	19.27	32.65	0.77	1306.03	25.81	21.92	19.22	17.18
Sex														
Male	7.22	0.33	880.25	35.94	31.47	27.92	25.06	28.75	1.03	1180.53	34.86	30.88	27.98	25.66
Female	7.35	0.34	883.13	36.17	31.72	28.22	25.41	30.05	1.09	1132.59	36.86	32.69	29.47	26.91
Transgender	8.57	0.30	344.07	10.43	7.67	5.40	3.12	0	0	0	0	0	0	0
Age														
0–14	6.93	0.37	737.93	32.49	27.93	24.21	21.25	41.46	1.82	1035.20	41.49	37.03	33.38	30.37
15–29	5.31	0.24	847.16	33.67	29.22	25.79	23.01	21.63	0.75	1163.30	34.14	30.22	27.35	25.00
30–59	8.17	0.36	993.24	39.71	35.33	31.87	29.09	25.33	0.87	1317.06	32.48	28.66	26.05	24.06
60+	11.64	0.47	953.51	36.65	32.15	28.68	25.84	37.16	1.10	1178.92	33.37	29.23	26.11	23.74
Religion														
Hindu	7.52	0.35	939.49	38.56	34.09	30.51	27.63	28.88	1.03	1169.49	35.51	31.43	28.37	25.92
Muslim	5.25	0.26	589.02	26.85	22.49	19.10	16.39	30.32	1.22	1082.32	39.06	34.98	31.92	29.46
Others*	9.35	0.32	867.01	27.22	22.78	19.46	16.90	35.12	1.13	1177.24	32.88	28.80	25.98	23.79
Social group														
STs	2.73	0.17	428.34	25.17	20.92	17.77	15.33	18.79	0.81	803.15	27.68	23.85	21.17	19.01
SCs	6.04	0.34	740.79	36.94	32.59	29.42	26.92	29.68	1.14	1115.62	34.38	30.52	27.77	25.64
OBCs	6.95	0.34	849.19	36.28	31.80	28.21	25.29	30.38	1.12	1181.39	38.42	34.26	31.04	28.45
Others	10.34	0.36	1133.42	36.82	32.33	28.66	25.73	31.09	0.99	1259.80	35.07	30.89	27.78	25.30
General education														
Illiterate	8.34	0.49	818.58	41.18	36.70	33.14	30.24	38.63	1.53	1085.49	36.13	31.97	28.79	26.19
Up to primary	5.67	0.29	707.85	30.83	26.39	22.93	20.22	27.46	1.07	1078.73	37.14	33.00	29.79	27.24
Secondary	8.98	0.36	1237.33	42.76	38.34	34.74	31.78	25.81	0.84	1500.09	36.85	32.97	30.29	28.20
Graduation and above	7.97	0.21	1287.96	28.91	24.38	20.81	17.96	19.85	0.52	1335.55	25.41	21.55	18.98	17.01
Wealth quintile														
Very poor	4.83	0.41	768.83	62.86	58.21	54.32	51.06	27.96	1.38	1063.93	46.77	42.59	39.24	36.48
Poor	6.18	0.32	714.71	36.77	32.32	28.82	25.85	24.97	0.97	1013.90	34.45	30.17	26.87	24.19
Average	8.75	0.33	814.81	30.47	26.06	22.59	19.86	37.63	1.14	1416.05	36.62	32.37	29.10	26.51
Rich	15.38	0.40	1269.47	33.05	28.58	24.96	22.06	28.99	0.68	1192.02	21.08	17.39	15.03	13.27
Very rich	14.82	0.21	1441.86	20.70	16.38	13.17	10.82	38.95	0.67	1912.17	24.45	20.80	18.54	16.86
Households size**														
Less than Average	7.98	0.32	842.32	31.43	27.01	23.60	20.91	30.94	0.87	1147.30	27.87	23.94	21.14	18.99
More than average	6.22	0.37	969.60	50.50	45.92	42.02	38.74	27.00	1.71	1172.43	71.14	66.43	62.27	58.60
Regions***														
North	6.62	0.25	906.54	32.01	27.58	24.02	21.21	30.82	0.99	1301.05	30.28	26.34	23.59	21.36
Northeast	2.24	0.12	380.27	18.50	13.80	10.31	7.98	12.57	0.51	1128.54	40.24	36.43	33.34	30.55
East	8.77	0.53	1296.07	64.88	60.29	56.33	52.92	38.52	1.77	1206.64	45.19	40.93	37.71	35.11
Central	3.34	0.20	506.92	28.49	24.19	21.08	18.65	28.16	1.27	1051.23	41.60	37.64	34.53	31.83
West	9.19	0.35	996.02	36.38	31.83	28.04	24.91	32.17	1.04	1221.09	32.28	28.36	25.47	23.33
South	9.72	0.36	797.15	28.02	23.62	20.27	17.67	20.15	0.55	1036.58	26.00	21.78	18.61	16.23

*Figures are based on the author's calculations from the NSSO 75th rounds*.

*Include remaining religions such as Christianity, Sikhism, Jainism, Buddhism, Zoroastrianism, and others.

**Households size is categorized into two categories viz. less than average and more than average.

***North: JandK, Himachal Pradesh, Punjab, Chandigarh, Uttarakhand, Haryana, Delhi, Rajasthan; Northeast: Sikkim, Arunachal Pradesh, Nagaland, Manipur, Mizoram, Tripura, Meghalaya, Assam; East: Bihar, West Bengal, Jharkhand, Odisha; Central: Utter Pradesh, Chhattisgarh, Madhya Pradesh; West: Gujrat, Daman, and Diu, Dadar and Nagar Haveli, Maharashtra, Goa; South: Andhra Pradesh, Karnataka, Lakshadweep, Kerala, Tamil Nadu, Pondicherry, Andaman and Nicobar, Telangana.

The OOP health expenditure on infectious diseases as a share of total consumption expenditure has been calculated as 0.34% in the study. On various demographic and socio-economic variables, it is relatively highest among individuals residing in rural areas (0.36%), females (0.34%), above 60+ (0.47%), and Hindus (0.35%). Among different social groups, it is 0.36% in other social groups. Among different education groups, illiterates (0.49%), among various economic groups, very poor wealth quintile (0.41%), among other households' size, more than average (0.37%), and among different geographical regions, eastern region (0.53%) spends a higher share of TCE on infectious diseases as compared to their respective counterparts in the country. On the other hand, OOP health expenditure on infectious diseases out of total consumption expenditure of infection-affected population has also been measured at different threshold levels in the study. The analysis illustrates that 36.04% of total consumption expenditure is spent on infectious diseases by the country's infection-affected population at the reporting level. It has been observed at different threshold levels: 31.59, 28.06, and 25.23%, with their corresponding levels as 5, 10, and 15%, respectively. The analysis shows that it declines with an increase in the infection's consumption expenditure from reporting to above threshold levels.

Similarly, India's overall average per capita OOP health expenditure on infectious diseases in outpatient care is INR 29.38. Furthermore, based on numerous socio-economic and demographic covariates, it is highest among urban areas (INR 32.65), among women (INR 30.05), among 0–14 age groups (INR 41.46), among other religious groups (INR 35.12), among different social groups (INR 31.09), among illiterate people (INR 38.63), among economically wealthy people (INR 38.95), among less than average households (INR 30.94), and people populated in the eastern region (INR 38.52) of India. At the same time, we calculated the average monthly per capita OOP expenditure of infection-affected population. Results show that INR 1,156.34 has been spent per month on outpatient care in India. Unlike inpatient care, it is highest in urban areas, rich wealth quintiles, and relatively weaker sections (socio-economically), such as female, 0–14 age groups, and Muslims, OBCs, illiterates' individuals than their corresponding counterparts in India. Furthermore, OOP health expenditure on infection as a percentage share of total consumption expenditure is 1.06% on outpatient care in the study. Whereas, at various socio-economic and demographic variables, it is highest among rural inhabitants (1.29%), females (1.09%), 60+ age groups (1.82%), Muslims (1.22%), scheduled castes (1.14%), illiterates (1.53%), very poor wealth quintiles (1.38%), more than average households' size (1.71%), and coming from eastern region (1.77%) than their respective counterparts in the analysis.

Furthermore, the percentage share of OOP health expenditure on infectious diseases as a share of total consumption expenditure of infection-affected populations has also been measured at different threshold levels in the study. The findings show that it is 35.82% of the total consumption expenditure of the country's infection-affected population at the reporting level. At the same time, it reduces proportionately with the increase in the threshold levels as 5, 10, and 15% in the analysis, respectively. Moreover, a similar trend of deterioration with an increase in threshold has been observed among various socio-economic and demographic variables. But the proportion has been found highest among patients inhabiting rural areas, females, 0–14 age groups, Muslims, OBCs, education up to primary levels, economically very poor people, accompanying large family size, and an inhabitant of the eastern region of India, respectively.

### Source of Finance to Cope With Out-of-Pocket Expenditure on Infectious Diseases

Table 4 shows that in the case of inpatient care, 86.4% of the infected population are using savings/income as a first source to finance the infection-derived expenditure in India. Simultaneously, the share of borrowings and other remaining coping strategies are only 8.4 and 5.0% in the country. Not using any coping strategies to finance health expenditure on infectious diseases has been reported in the study, which constituted only a 0.3% share of the infection-affected population in the country. Furthermore, concerning the individuals' residence and sex, the percentage of savings/income is highest among people residing in the urban area (89.1%) and female (87.5%) patients. In comparison, borrowings are significantly prevalent among rural (10.1%) and male (9.0%) patients in India. In the patients' age, both savings/income (87.2%) and borrowings (9.2%) are highly employed as a source of finance by the 0–14 age group in the study. While concerning religion, the share of both sources of finance for coping is relatively less among Muslim people, but other residual coping strategies are highest in the study.

**Table 4:** Socio-economic and demographic covariates in the source of finance to cope with out-of-pocket (OOP) expenditure on infectious diseases in India (2017–18).

**Variables**	**Inpatient**				**Outpatient**			
	**Savings/** **Income**	**Borrowings**	**Others** ^**#**^	**Not reported any source of finance**	**Savings/** **Income**	**Borrowings**	**Others** ^**#**^	**Not reported any source of finance**
All India	86.4	8.4	5.0	0.3	92.9	1.5	2.8	3.0
	(85.9–87.9)	(7.9–9.4)	(4.4–5.8)	(0.1–0.4)	(91.9–93.9)	(1.1–2.0)	(2.1–3.5)	(2.4–3.6)
Place of residence								
Rural	85.6	10.1	5.1	0.3	92.4	1.8	2.8	3.4
	(84.3–86.9)	(9.1–11.2)	(4.2–6.0)	(0.03–0.5)	(91.2–93.6)	(1.1–2.4)	(2.0–3.5)	(2.6–4.1)
Urban	89.1	5.9	5.0	0.3	94.2	1.0	2.9	2.1
	(87.8–90.6)	(5.1–6.9)	(3.9–6.1)	(0.2–0.5)	(92.6–95.9)	(0.4–1.6)	(1.6–4.3)	(1.3–3.0)
Sex								
Male	86.1	9.0	5.0	0.3	93.2	1.5	2.9	2.7
	(84.8–87.8)	(7.9–10.3)	(4.0–6.0)	(0.1–0.6)	(91.8–94.6)	(0.8–2.2)	(1.8–3.6)	(1.9–3.5)
Female	87.5	8.2	5.2	0.2	92.7	1.6	2.7	3.3
	(86.2–88.7)	(7.3–9.1)	(4.3–6.1)	(0.1–0.3)	(91.3–94.1)	(0.9–2.3)	(1.9–3.6)	(2.4–4.2)
Transgender	1.0	0	0	0	0	0	0	0
	0	0	0	0	0	0	0	0
Age								
0–14	87.2	9.2	4.5	0.6	94.1	1.3	2.2	2.4
	(85.6–88.7)	(8.0–10.5)	(2.9–4.5)	(0.1–1.2)	(92.8–94.1)	(0.7–1.9)	(1.3–3.1)	(1.7–3.2)
15–29	85.5	9.0	6.0	0.1	91.4	2.2	4.1	2.4
	(83.4–88.2)	(7.3–10.7)	(4.3–7.8)	(0.05–0.2)	(88.8–94.1)	(0.8–3.5)	(1.9–6.2)	(1.4–3.4)
30–59	87.1	8.4	4.7	0.1	93.7	1.1	2.2	3.7
	(85.5–89.0)	(7.2–9.9)	(3.6–6.1)	(0.01–0.2)	(91.9–95.4)	(0.3–1.9)	(1.3–3.2)	(2.4–5.1)
60+	87.0	6.3	8.0	0.1	88.3	2.7	4.6	4.8
	(84.6–89.4)	(4.8–8.2)	(6.0–10.0)	(0.02–0.2)	(84.0–92.7)	(0.5–4.9)	(1.5–7.8)	(2.3–7.3)
Religion								
Hindu	87	8.6	4.7	0.2	93.1	1.4	2.7	3.0
	(86.0–88.2)	(8.0–9.6)	(4.1–5.7)	(0.1–0.3)	(92.0–94.3)	(0.9–1.9)	(1.9–3.4)	(2.4–3.7)
Muslims	85.6	7.9	6.2	0.6	91.8	2.8	3.6	2.2
	(83.0–88.6)	(5.8–10.2)	(4.6–8.1)	(−0.2–1.5)	(89.1–94.5)	(0.9–4.6)	(1.8–5.3)	(1.1–3.4)
Others*	87.2	8.7	4.3	0.2	93.4	0.6	2.7	4.6
	(83.8–90.6)	(5.7–11.9)	(2.8–5.9)	(0.1–0.3)	(90.2–96.5)	(−0.03–1.2)	(0.5–4.8)	(1.8–7.4)
Households size**								
Less than average	86.5	8.5	5.4	0.2	91.8	0.2	3.3	3.5
	(85.4–87.8)	(7.7–9.4)	(4.6–6.5)	(0.1–0.3)	(90.4–93.1)	(1.1–2.4)	(2.4–4.3)	(2.7–4.4)
More than average	87.4	8.7	4.0	0.3	95.0	1.1	1.9	2.1
	(85.8–89.2)	(7.4–10.4)	(3.1–4.9)	(−0.001–0.8)	(93.7–96.3)	(0.4–1.8)	(1.0–2.8)	(1.5–2.8)
Social groups								
STs	88.1	6.2	5.5	0.4	88.2	0.4	4.2	7.3
	(85.8–90.5)	(4.6–7.8)	(4.0–7.4)	(0.2–0.7)	(84.5–91.8)	(0.05–0.7)	(1.8–6.6)	(4.4–10.1)
SCs	86.3	10.2	4.0	0.2	92.7	1.4	1.9	4.3
	(84.4–88.4)	(8.5–12.1)	(3.1–5.0)	(0.01–0.4)	(90.4–94.9)	(0.4–2.3)	(0.8–2.9)	(2.5–6.1)
OBCs	86.1	9.7	4.5	0.2	93.9	1.4	2.7	2.2
	(84.6–87.8)	(8.7–11.1)	(3.7–6.1)	(0.03–0.3)	(92.5–95.3)	(0.8–2.0)	(1.7–3.8)	(1.4–2.9)
Others	88	6.1	5.7	0.5	93.0	2.3	3.2	2.1
	(86.1–89.8)	(5.0–7.5)	(4.6–7.3)	(0.03–1.0)	(90.9–95.1)	(1.0–3.6)	(1.7–4.8)	(1.3–2.9)
General education								
Illiterate	84.5	10.1	5.5	0.5	92.2	1.3	3.0	3.7
	(82.6–86.8)	(8.6–11.8)	(4.2–7.1)	(0.04–0.9)	(90.5–94.0)	(0.7–1.9)	(1.8–4.2)	(2.5–4.8)
Up to primary	88	8.1	4.2	0.2	93.6	1.7	2.3	2.8
	(86.8–89.2)	(7.3–9.2)	(3.7–5.2)	(0.09–0.3)	(92.2–95.0)	(0.9–2.6)	(1.5–3.1)	(1.9–3.6)
Secondary	86.6	8.4	5.2	0.2	93.3	1.3	3.8	2
	(84.6–89.0)	(6.8–10.2)	(3.8–6.8)	(−0.1–0.4)	(90.3–96.2)	(−0.04–2.6)	(1.2–6.4)	(1.1–2.9)
Graduation and above	90.6	3.8	5.6	0.1	91.2	2.5	3.3	3.4
	(86.3–95.1)	(2.2–5.7)	(1.6–9.9)	(−0.05–0.3)	(86.4–95.9)	(−0.7–5.8)	(0.7–5.8)	(0.7–6.1)
Wealth quintile								
Very poor	85.6	9.3	5.3	0.3	91.5	1.8	3.2	3.7
	(83.9–87.5)	(7.9–10.8)	(4.4–6.6)	(−0.1–0.7)	(89.8–93.1)	(1.0–2.5)	(2.0–4.3)	(2.7–4.7)
Poor	84.2	11	5.4	0.2	94.8	1.5	2	1.8
	(81.9–86.7)	(9.4–12.8)	(3.6–7.6)	(0.05–0.4)	(93.2–96.4)	(0.5–2.5)	(1.0–2.9)	(1.0–2.7)
Average	88.1	8.2	3.7	0.5	94.3	1.4	2.8	2.1
	(86.2–90.1)	(6.7–10.0)	(2.8–5.0)	(0.1–0.9)	(92.4–96.3)	(0.3–2.4)	(1.4–4.3)	(1.2–3.1)
Rich	90.5	5.7	4.0	0.2	95.7	1.3	1.5	2.5
	(88.8–92.5)	(4.5–7.4)	(2.8–5.3)	(0.03–0.3)	(93.6–97.8)	(−0.4–2.9)	(5.8–2.4)	(1.0–4.1)
Very rich	91.1	2.5	6.1	0.2	89.2	0.4	5.7	5.5
	(89.0–93.3)	(1.6–3.8)	(4.4–8.2)	(−0.01–0.3)	(82.4–96.0)	(−0.2–1.0)	(0.05–11.3)	(1.1–9.9)
Regions***								
North	89.6	7.6	2.6	0.4	95.4	1.3	0.9	3.3
	(87.6–91.8)	(6.0–9.5)	(1.6–3.8)	(0.01–0.7)	(93.3–97.5)	(0.2–2.4)	(0.2–1.7)	(1.4–5.2)
Northeast	95.1	2.6	2.0	0.3	89.6	1.2	1.2	8.1
	(93.5–96.7)	(1.3–3.9)	(1.0–3.1)	(0.1–0.4)	(82.5–96.6)	(−0.4–2.8)	(−0.3–2.8)	(1.3–14.8)
Central	87.1	8.1	5.0	0.3	93.6	1.6	3.4	1.4
	(84.7–89.6)	(6.1–10.4)	(4.0–6.2)	(−0.004–0.6)	(91.6–95.6)	(0.7–2.6)	(1.8–5.0)	(0.6–2,2)
East	88.2	6.0	5.6	0.6	92.3	0.7	4.0	3.0
	(86.5–90.3)	(4.8–7.2)	(4.4–7.1)	(−0.1–1.4)	(90.4–94.3)	(0.4–1.1)	(2.4–5.5)	(1.8–4.3)
West	92.4	5.2	2.3	0.1	92.7	2.7	1.6	3.0
	(90.7–94.5)	(3.5–7.0)	(1.6–3.1)	(0.02–0.2)	(89.8–95.6)	(0.7–4.7)	(0.03–3.2)	(1.5–4.5)
South	81.0	13.0	7.2	0.1	91.2	1.7	2.7	5.2
	(79.0–83.2)	−11.6	(5.5–9.1)	(0.04–0.2)	(88.9–93.5)	(0.7–2.8)	(1.7–3.7)	(3.4–7.1)

*Figures are based on the author's calculations from the NSSO 75th rounds*.

*Values in parentheses are 95% confidence interval*.

*^#^Includes the remaining source of finance such as the sale of physical assets, contributions from friends and relatives, and other sources*.

^*^Include remaining religions such as Christianity, Sikhism, Jainism, Buddhism, Zoroastrianism, and others.

^**^Household size is categorized into two categories viz. less than average and more than average.

^***^North: JandK, Himachal Pradesh, Punjab, Chandigarh, Uttarakhand, Haryana, Delhi, Rajasthan; Northeast: Sikkim, Arunachal Pradesh, Nagaland, Manipur, Mizoram, Tripura, Meghalaya, Assam; East: Bihar, West Bengal, Jharkhand, Odisha; Central: Utter Pradesh, Chhattisgarh, Madhya Pradesh; West: Gujrat, Daman, and Diu, Dadar and Nagar Haveli, Maharashtra, Goa; South: Andhra Pradesh, Karnataka, Lakshadweep, Kerala, Tamil Nadu, Pondicherry, Andaman and Nicobar, Telangana.

Among social categories, savings/income are employed by STs (88.1%), and borrowings are used mainly by SCs (10.2%) in India. Furthermore, savings/income (90.6%) are highly used by educated people suffering from infectious diseases in the analysis. Still, the dependency on borrowing (10.1%) is relatively highest among illiterate people in the country. Among different economic statuses, the share of savings/income is highest among wealthy people, but the poor are more dependent on borrowings in the study. The analysis shows that savings/income and borrowings are the leading sources of finance for coping among individuals related to large family size. Finally, savings/income with a 95.1% share is the top strategy for dealing in the north-east region. In contrast, individuals from the southern part have a larger share of borrowing to finance their inpatient care expenditure in India.

In outpatient care, savings/income with a 92.9% share is the leading strategy to finance health-care expenditure in India. However, it has been seen that the percentage of borrowings is not very much significant in the study. But as a source of finance, borrowings and other coping strategies contribute only 1.5 and 2.8% to the total share of expenditure on infectious diseases in the country. The findings also illustrate that nearly 3.0% of people suffering from infection did not report any source of finance in the analysis. Based on various socio-economic variables, savings/income is primely used by urban (94.2%) inhabitants and male (93.2%) patients in India. It is highest among different age groups among 0–14 with a 94.1% share. Although the overall percentage of borrowings is less significant among all coping strategies, dependence has been higher among the aged, Muslims, educated, and very poor. Again, it has been seen that 93.9% OBCs are dependent on savings/income while not reporting any source of finance for coping and other remaining strategies are highest among STs in India. The share of savings/income is highest among educated people in the country on educational background. Finally, the percentage of savings/income is highest in the northern region. At the same time, the share of people who did not report any copying strategies is highest in the north-eastern part of India.

## Discussion and Conclusion

Overall, the results indicate a significant existence of infectious diseases that are still a big threat to India's public health. Although several horizontal and vertical policy initiatives to cure, control and eradicate infectious diseases have been taken into account by the governments, but could not provide any landmark changes in this regard. It has been perceived that these infectious diseases are not easily controllable until the worse surroundings, such as the lack of cleanliness, open defecation, and many other associated factors, are addressed. In inpatient and outpatient cases, infectious diseases are significantly prevalent among rural areas, females, transgender, children (0–14 age group), aged persons (60+ age group), SCs, non-Hindu communities, and illiterate people in India. In the analysis, the relatively lower percentage of poor people suffering from infectious diseases does not illustrate their better health conditions. Still, it reflects their un-reportability to health-care facilities than economically prosperous people. This results from their insufficient financial resources and fulfills this notion that accessibilities of health-care facilities are still far from the reach of these economically marginalized sections of Indian societies.

Furthermore, the analysis shows that OOP expenditure on infectious diseases is comparatively high in outpatient care. Per patient OOP expenditure on contagious diseases has been found lower among most socio-economically vulnerable groups, such as rural inhabitants, transgender people, Muslims, scheduled tribes, illiterates, educated up to primary level, and poor wealth quintile in India. Also, a declining trend of average per capita OOP expenditure with an increase in the thresholds level has been seen. The result further elaborates that people rely more on savings/income to cope with infectious diseases. Still, borrowings are employed significantly to manage inpatient care than outpatient care in India. Also, socio-economically deprived people, such as rural people, SCs, illiterate, and very poor, are more dependent on borrowing than others. This fact exaggerates the belief that these people are still reliant on borrowings to attain basic needs, including health, due to their improper inclusion in the country's main streams. Furthermore, an interesting fact regarding education status and different sources of finance for coping has been observed in the study. The analysis states a positive impact on the use of savings/income with an increase in the individuals' education level.

This study concludes that infectious diseases are still a significant threat to public health in India. Various life-threatening, long-lasting contagious diseases such as smallpox, cholera, plague, dengue, and flu pandemic, have been controlled, cured, and eradicated through numerous vertical and horizontal disease control programmes. But several re-emerging infectious diseases are increasing the challenges of health care, treatment behavior, health-care costs, and source of finance for coping in the country. The global emergence of COVID-19 has challenged humanity's survival in India and worldwide (38). Since its emergence, the continuously rising number of active cases has warned the world of its death toll. With the government's imposition of social distancing through lockdown, the only way to prevent such pandemic has radically broken the entire social structure, cultural values, and economic systems (15, 16). This evidence hypothesizes that socially and economically vulnerable sections of societies become worse when they suffer from such contagious diseases. It increases their burden of health care through OOP health expenditure and reduces their productive efficiency during the spell of ailments. For instance, in the study, SCs depend more on borrowings to finance infectious diseases than other social groups in India. The idea of dependency on borrowings to fund health-care expenditure communicates that these deprived sections are still at the mercy of their masters and very far from the mainstream of the country.

Additionally, to curtail the share of OOP expenditure on health care, an increase in the percentage of GDP on health care and universalisation of insurance coverage is the need of the hour (36). According to the budget estimates for the fiscal year 2018, around 1.3% of India's GDP has been spent on public health, whereas it is a minimum of 6–7% of GDP in most European countries. Only 12% of the urban and 13% of the rural population are under the protection coverage through Rashtriya Swasthya Bima Yojana (RSBY) in the country (39). Though various health insurance schemes have been launched by the country's union and state governments, people without insurance coverage are still considerable due to the improper implementations. As a result, nearly 86% of the rural population and 82% of the urban population were not covered under any public or private scheme of health expenditure support in India (39). To fulfill the objectives of maximum population coverage, another plan, “Ayushman Bharat,” PM-JAY the world's largest government-funded health-care scheme, was launched by the Government of India in 2018 under National Health Policy 2017. The scheme provides a health cover of Rs. 5 lakhs per family per year, specifically targeted 10.74 crore poor and vulnerable families (~50 crore beneficiaries) (40). The plan ensures financial protection against catastrophic health expenditure and access to affordable and quality health care for all country's citizens (41).

In addition, for ensuring the quality of health-care services, the Government of India had initiated the “National Digital Health Mission (NDHM)” in 2021. The mission will create a national digital health ecosystem to support universal health coverage in an efficient, accessible, inclusive, affordable, timely, and safe manner. The system will provide a wide range of data, information, and infrastructure services to the people (41, 42). However, these initiatives have been holistically launched for ensuring better health facilities, but it is early to make any prediction regarding its outcomes; hopefully, the time will define it over the passing of a few more years.

Also, spreading awareness among people about cleanliness, sanitation, and free from open defecation has positively impacted the country's individuals' health. The findings tabled the fact that Swachh Bharat Mission-Gramin (SBM-G) has reduced the number of diarrheal cases. They avoided more than 14 million Disability-Adjusted Life Years (DALYs) between 2014 and October 2019 (43, 44). Recently, to prevent the community transmission of the coronavirus disease, propagation of awareness through multiple media platforms changed the public attitudes and behavior toward susceptible people to this contagious disease (15, 45). The awareness campaigns not only reduced the chances of contact with coronavirus but also encouraged the asymptomatic individuals to conduct health protocols, such as self-isolation and social distancing, in the country (45). Therefore, the result deliberates the need for proper implementations of policy initiatives against ailments, outcome-oriented implementation of health-care schemes among targeted population, ensuring quality of public health-care system and its expansion nearer to the people's doorsteps, immunisations/vaccinations drives, subsidized medical facilities to vulnerable sections, and awareness programmes against unhygienic conditions in the country.

## Data Availability Statement

The datasets presented in this study can be found in online repositories. The names of the repository/repositories and accession number(s) can be found below: www.mospi.gov.in.

## Author Contributions

BR conceived the idea and prepared the initial draft of the manuscript. BR and RT performed the statistical analysis. RT revised the manuscript. Both authors read and approved the final manuscript.

## Funding

The authors acknowledge the financial support of the Ministry of Human Resource Development (MHRD), Government of India for conducting this study, under the Scheme for Promotion of Academic and Research Collaboration (SPARC) (Project Code: P1173).

## Conflict of Interest

The authors declare that the research was conducted in the absence of any commercial or financial relationships that could be construed as a potential conflict of interest.

## Publisher's Note

All claims expressed in this article are solely those of the authors and do not necessarily represent those of their affiliated organizations, or those of the publisher, the editors and the reviewers. Any product that may be evaluated in this article, or claim that may be made by its manufacturer, is not guaranteed or endorsed by the publisher.

## Abbreviations

OOP: out-of-pocket
INR: Indian National Rupee
LMICs: low- and middle-income countries
SCs: scheduled castes
STs: scheduled tribes
OBCs: other backward classes
TCE: total consumption expenditure
NSSO: National Sample Survey Organization
HIV/AIDS: human immune virus/acquired immune deficiency syndrome
GDP: gross domestic product
NHP: national health policy.
